# The association between menstrual cycle characteristics and cardiometabolic outcomes in later life: a retrospective matched cohort study of 704,743 women from the UK

**DOI:** 10.1186/s12916-023-02794-x

**Published:** 2023-03-20

**Authors:** Kelvin Okoth, William Parry Smith, G. Neil Thomas, Krishnarajah Nirantharakumar, Nicola J. Adderley

**Affiliations:** 1 Institute of Applied Health Research, IOEM Building, University of Birmingham, University of Birmingham, Edgbaston, Birmingham, B15 2TT UK; 2 Institute of Metabolism and Systems Research, University of Birmingham, Birmingham, UK; 3 Centre for Endocrinology, Diabetes and Metabolism, Birmingham Health Partners, Birmingham, UK

**Keywords:** Menstrual cycle, Cardiovascular disease, Hypertension, Type 2 diabetes mellitus

## Abstract

**Background:**

Female reproductive factors are gaining prominence as factors that enhance cardiovascular disease (CVD) risk; nonetheless, menstrual cycle characteristics are under-recognized as a factor associated with CVD. Additionally, there is limited data from the UK pertaining to menstrual cycle characteristics and CVD risk.

**Methods:**

A UK retrospective cohort study (1995–2021) using data from a nationwide database (The Health Improvement Network). Women aged 18–40 years at index date were included. 252,325 women with history of abnormal menstruation were matched with up to two controls. Two exposures were examined: regularity and frequency of menstrual cycles; participants were assigned accordingly to one of two separate cohorts. The primary outcome was composite cardiovascular disease (CVD). Secondary outcomes were ischemic heart disease (IHD), cerebrovascular disease, heart failure (HF), hypertension, and type 2 diabetes mellitus (T2DM). Cox proportional hazards regression models were used to derive adjusted hazard ratios (aHR) of cardiometabolic outcomes in women in the exposed groups compared matched controls.

**Results:**

During 26 years of follow-up, 20,605 cardiometabolic events occurred in 704,743 patients. Compared to women with regular menstrual cycles, the aHRs (95% CI) for cardiometabolic outcomes in women with irregular menstrual cycles were as follows: composite CVD 1.08 (95% CI 1.00–1.19), IHD 1.18 (1.01–1.37), cerebrovascular disease 1.04 (0.92–1.17), HF 1.30 (1.02–1.65), hypertension 1.07 (1.03–1.11), T2DM 1.37 (1.29–1.45). The aHR comparing frequent or infrequent menstrual cycles to menstrual cycles of normal frequency were as follows: composite CVD 1.24 (1.02–1.52), IHD 1.13 (0.81–1.57), cerebrovascular disease 1.43 (1.10–1.87), HF 0.99 (0.57–1.75), hypertension 1.31 (1.21–1.43), T2DM 1.74 (1.52–1.98).

**Conclusions:**

History of either menstrual cycle irregularity or frequent or infrequent cycles were associated with an increased risk of cardiometabolic outcomes in later life. Menstrual history may be a useful tool in identifying women eligible for periodic assessment of their cardiometabolic health.

**Supplementary Information:**

The online version contains supplementary material available at 10.1186/s12916-023-02794-x.

## Background

Cardiovascular disease (CVD) is a major public health burden and remains the leading cause of mortality in women accounting for 35% of the total deaths worldwide based on estimates from the global burden of disease study [1, 2]. Recent literature reviews and consensus statements from professional societies in the US and Europe have highlighted the association between female reproductive factors and risk of CVD in later life [3–6]. However, menstrual cycle history and its relation to CVD was not included despite evidence of its association with CVD risk [7–9].

The menstrual life course begins at menarche and ends at menopause. The regulation of menstrual cycles involves an intricate balance between hypothalamic, pituitary, and gonadal axis hormones. A disruption of this balance may result in changes in menstrual characteristics that may affect one or more of four menstrual cycle domains: frequency, regularity, duration, or volume of flow [10]. The years immediately after menarche and the menopausal transition period are characterized by irregular and unstable menstrual cycles [11]. When menstrual cycles are stable, a typical menstrual period will last for 3 to 5 days, while the average menstrual cycle will last for 28 days (range 21–35 days) [11]. Long or irregular menstrual cycles are associated with cardiovascular risk factors including hyperinsulinemia and dyslipidemia, hypertension, and diabetes mellitus [12, 13]. The American College of Obstetricians and Gynaecologists recommends the inclusion of menstrual cycle history as a vital sign to improve the timely identification of potential adverse health outcomes in later life [14]. However, the management of abnormal menstruation focuses primarily on addressing associated infertility challenges with other potential longer-term risks underappreciated.

The UK provides universal health care to all its residents. The first point of call for UK women with clinically significant changes in the menstrual cycle patterns will be the primary care practice. The present study will harness electronic health data from UK primary care to shed more light on the association between menstrual cycle characteristics and risk of cardiometabolic outcomes in the future.

## Methods

### Study design

A population-based retrospective cohort study was conducted to evaluate the association between menstrual cycle characteristics and long-term risk of cardiometabolic outcomes. Only domains relating to the regularity and frequency of menstrual cycle were used in the present study (Additional file: Table S1) [10]. Therefore, two study cohorts were created. The first cohort was composed of women with irregular or no menstrual cycle (exposed group) and matched controls from the general population without a history of irregular menstrual cycles. The second cohort was composed of women with infrequent or frequent menstrual cycles (exposed group) and matched controls from the general population without a history of infrequent or frequent menstrual cycles. The study period was 1 January 1995 to 31 December 2021. The rates of cardiometabolic outcomes were compared in the exposed and control groups.

### Data source

IQVIA Medical Research Data (IMRD) incorporates data from The Health Improvement Network (THIN), a Cegedim database. Reference made to THIN is intended to be descriptive of the data asset licensed by IQVIA. The proposed study used de-identified data provided by patients as a part of their routine primary care. IMRD-UK (formerly THIN) is a nationwide UK-based database containing anonymized electronic health records contributed by 787 general practices. Registered practices contributing to the database are representative of the UK population [15, 16]. Participating practices collect patient data using an electronic health records software system known as the Vision software.

### Practice eligibility criteria

Practices were eligible for inclusion from the later of the date on which the practice met acceptable mortality reporting (a quality assurance standard) or 1 year after the practice began to use the Vision software system [17].

### Study population

The study population was composed of women aged 18–40 years at baseline. Participants entered the study at the latest of their 18th birthday, study start date (1 January 1995), or 1 year after joining the practice (to ensure sufficient time for recording of baseline information).

### Exposure

The coding of diagnoses and other health-related care processes in UK primary care is based on the Read code clinical terminology (computable phenotype) [18]. The exposures of interest were identified by the presence of a diagnostic Read code describing menstrual cycle irregularity or frequent or infrequent cycles as reported in primary care. Menstrual cycle characteristic self-report has been validated in other studies and is regarded as reliable [19, 20]. Where a patient had a diagnostic record for both irregularity and frequent or infrequent menstrual cycles, exposure status was assigned to the first ever recorded domain. Characteristics relating to the regularity of the menstrual cycle defined a composite exposure that included irregular cycles, amenorrhea, menometrorrhagia, and metropathia haemorrhagica. Attributes relating to menstrual cycle frequency defined a composite exposure that included too frequent (polymenorrhea, epimenorrhea) or infrequent (oligomenorrhea) cycles. Details are provided in Additional file: Table S2. Women with the exposure of interest were matched with up to two women without a record of the exposure (controls), randomly selected from a pool of eligible women. The exposed and unexposed groups were matched by age (± 1 year) and general practice. Women with a record of other menstrual related conditions including intermenstrual bleeding, menstrual disorders, and complications of duration or volume of flow were excluded from the study.

### Follow-up period

For newly (incident) diagnosed exposures (irregular cycles and frequent or infrequent cycles), the date of diagnosis served as the index date. For patients with a pre-existing record relating to complications in the regularity or frequency of menstrual cycles, the date the patient became eligible to participate in the study served as the index date. To mitigate immortal time bias, exposed patients were assigned the same index date as their corresponding controls and matched on this date [21]. Each exposed and matched control participant contributed follow-up time from the index to the exit date. The exit date was the earliest of (i) the outcome, (ii) death, (iii) study end date, and (iv) date of leaving the general practice or when the general practice stopped contributing to the database.

### Outcomes

The primary outcome was the incident diagnosis of cardiovascular disease, a composite of ischemic heart disease, heart failure, or cerebrovascular disease (stroke or transient ischemic attack). Secondary outcomes were the cardiovascular conditions separately, hypertension and type 2 diabetes mellitus. Participants with a diagnosis of the outcome of interest at baseline were excluded from the corresponding crude and adjusted regression analysis. Outcomes were identified using the relevant Read codes. The Read codes used in the present study were selected using a method comparable to that proposed by Davé and Peterson and Watson et al. [22, 23]. First, a list of pertinent medical terms associated with the outcomes was compiled. Using the medical terms identified in the first step, the description, and numeric fields (columns) of the Read code dictionary were searched for relevant diagnostic codes related to the outcomes of interest. Third, we compared the codes identified in the previous step with codes published in online Read code repositories (caliberresearch.org, clinicalcodes.org, Cambridge code lists index) [24–26], as well as codes published in supplementary material of existing literature [27]. Finally, we consulted with UK clinicians to determine the final set of codes to be used in the study. All the outcomes in this study are included in the United Kingdom’s Quality and Outcomes Framework (QOF), a pay-for-performance system. The QOF was established to improve chronic disease management by financially rewarding primary care practices for providing interventions associated with better health outcomes. Chronic conditions falling under the QOF domains are well documented in UK general practices. Validation studies demonstrate that the prevalence of chronic diseases in THIN databases is comparable to national estimates [15, 28, 29].

### Study covariates

The following potential confounders were included in the study: sociodemographic characteristics (age and Townsend index of deprivation), lifestyle characteristics (body mass index [BMI], smoking status, alcohol use), medical characteristics (current lipid medication, connective tissue disorders, migraine), and reproductive factors (current oral contraceptive pills use [COC], preeclampsia, gestation diabetes mellitus, pregnancy loss, pre-term delivery, polycystic ovary syndrome [PCOS], endometriosis, pelvic inflammatory disease and uterine fibroids). Age was calculated at index date. The Townsend deprivation index is a measure of material deprivation derived from census data and linked to residential area [30]. The Townsend deprivation index is computed using the following domains: unemployment as a percentage of economically active individuals aged 16 and older, car ownership as a percentage of all households, home ownership as a percentage of all households, and overcrowding. BMI was calculated as weight divided by height in meters squared and categorized using WHO criteria (< 18.5, 18.5–24.9. 24–29.9, and > 30 kg/m^2^) [31]. Smoking (non-smokers, current smokers, ex-smokers) and alcohol use (non-drinkers, drinkers with excess, drinker without excess, ex-drinker) were self-reported. Self-reported smoking status and self-reported alcohol use are reliably recorded in THIN database [32, 33]. Current lipid medication was defined as the prescription of lipid medication within 60 days of cohort entry. Connective tissue disorders included rheumatological diseases (systemic lupus erythematosus, polymyositis, mixed connective tissue disease, polymyalgia rheumatica, moderate to severe rheumatoid arthritis). Current combined oral contraceptive pills use was defined as contraceptive use within 1 year of cohort entry. For each of the covariates, the latest record of the variable prior to study entry was used.

### Analysis

Participant characteristics at baseline were reported using median (IQR) for continuous variables and counts (%) for categorical variables. The crude incidence rates of cardiometabolic outcomes were estimated for each exposure group. Unadjusted and adjusted Cox proportional hazard models were used to derive hazard ratios (HR) and 95% confidence intervals (95% CI) for the associations between menstrual cycle characteristics (regularity or frequency) and incident cardiometabolic outcomes. In the multivariable models, adjustments were made for age, BMI, Townsend deprivation quintiles, smoking status, COC pills use, lipid-lowering drug use, alcohol use, connective tissue disorders, reproductive complications, and migraine. A separate category called missing was created for categorical data with missing data and incorporated in the regression analysis. For each model, the proportional hazards assumption was evaluated using the Schoenfeld residual test and graphical confirmation using the log–log survival curves.

### Sensitivity analysis

We performed several sensitivity analyses on the primary outcome to evaluate the robustness of our findings. Women with several reproductive characteristics, including polycystic ovary syndrome, amenorrhea, endometriosis, fibroids, and current contraceptive use, were excluded to evaluate whether these conditions drove any observed associations. We also examined, separately, the association between frequent or infrequent menstrual cycles and their relationship to cardiometabolic outcomes. Additionally, we evaluated any potential interaction between abnormal menstrual cycles (irregular and frequent or infrequent) and lifestyle characteristics (body mass index, smoking and alcohol consumption).

A two-tailed *p*-value of 0.05 was considered statistically significant. All analyses were conducted using Stata SE version 17.0.

## Results

Additional file: Figure S1 presents the study participants flow chart. There were 704,743 patients in the present study including 215,378 with a history of irregular menstrual cycles and 36,947 with a history of frequent or infrequent menstrual cycles (Table 1). By design, the median age of women in the exposed and unexposed groups was similar (approximately 27 years). Compared to women who had regular cycles, women with irregular menstrual cycles were more likely to be obese (15.4% versus 10.9%), be current smokers (24.9% versus 21.1%), be in the most deprived Townsend quintile (14.8% versus 13.1%), have migraine (27.8% versus 21.3%), have a current prescription for COC pills before cohort entry (30.6% versus 27.0.%), have a history of miscarriage (9.2% versus 6.2%), and have a diagnosis of polycystic ovary syndrome (5.6% versus 1.7%). A similar pattern in baseline differences was present in the group examining women with frequent or infrequent menstrual cycles compared to women with menstrual cycles of normal frequency.

**Table 1:** Baseline characteristics by menstrual characteristics status

Characteristics	Irregular cycles (*N* = 215 378)	Regular cycles (*N* = 386 825)	Frequent/infrequent cycles (*N* = 36 947)	Normal cycle frequency (*N* = 65 593)
	***n* (%)**	***n* (%)**	***n* (%)**	***n* (%)**
***Age****; median (IQR)*	27.5 (22.1–33.2)	27.2 (22.0–32.7)	27.5 (21.8–33.8)	27.4 (21.9–33.4)
***Townsend deprivation quintile***				
1 (least deprived)	36,877 (17.1)	71,109 (18.4)	6913 (18.7)	12,912 (19.7)
2	33,111 (15.4)	61,786 (16.0)	5916 (16.0)	10,949 (16.7)
3	38,970 (18.1)	70,821 (18.3)	6765 (18.3)	12,014 (18.3)
4	39,909 (18.5)	68,329 (17.7)	6630 (17.9)	11,310 (17.2)
5 (most deprived)	31,911 (14.8)	50,707 (13.1)	5054 (13.7)	8171 (12.5)
Missing	34,600 (16.1)	64,073 (16.6)	5669 (15.3)	10,237 (15.6)
***BMI categories in kg/m***^***2***^				
18.5–25	87,884 (40.8)	157,658 (40.8)	14,245 (38.6)	26,492 (40.4)
< 18.5	10,107 (4.7)	15,996 (4.1)	1561 (4.2)	2589 (4.0)
25–30	37,110 (17.2)	61,591 (15.9)	6233 (16.9)	10,537 (16.1)
> 30	33,194 (15.4)	41,996 (10.9)	6234 (16.9)	6925 (10.6)
Missing	47,083 (21.9)	109,584 (28.3)	8674 (23.5)	19,050 (29.0)
***Smoking status***				
Non-smokers	121,477 (56.4)	220,926 (57.1)	20,922 (56.6)	37,065 (56.5)
Current smokers	53,576 (24.9)	81,706 (21.1)	8629 (23.4)	13,766 (21.0)
Ex-smokers	23,951 (11.1)	38,887 (10.1)	4012 (10.9)	6434 (9.8)
Missing	16,374 (7.6)	45,306 (11.7)	3384 (9.2)	8328 (12.7)
***Alcohol status***				
Non-drinker	40,821 (19.0)	65,386 (16.9)	6469 (17.5)	10,345 (15.8)
Drinker with excess	4720 (2.2)	5887 (1.5)	737 (2.0)	932 (1.4)
Drinker no excess	111,018 (51.6)	192,483 (49.8)	18,887 (51.1)	32,934 (50.2)
Ex-drinker	2329 (1.1)	3397 (0.9)	348 (0.9)	580 (0.9)
Missing	56,490 (26.2)	119,672 (30.9)	10,506 (28.4)	20,802 (31.7)
***Current lipid medication***	456 (0.2)	527 (0.1)	87 (0.2)	79 (0.1)
***Connective tissue disorders***	999 (0.5)	1498 (0.4)	168 (0.5)	289 (0.4)
***Migraine***	59,873 (27.8)	82,328 (21.3)	10,550 (28.6)	13,877 (21.2)
***Reproductive factors***				
Current combined oral contraceptive pills	65,820 (30.6)	104,274 (27)	10,266 (27.8)	17,339 (26.4)
Polycystic ovary syndrome	11,970 (5.6)	6448 (1.7)	3755 (10.2)	1017 (1.6)
Pelvic inflammatory disease	6283 (2.9)	6941 (1.8)	1100 (3.0)	1193 (1.8)
Endometriosis	2353 (1.1)	3730 (1.0)	417 (1.1)	639 (1.0)
Fibroids	698 (0.3)	1268 (0.3)	144 (0.4)	210 (0.3)
Miscarriage	19,745 (9.2)	23,954 (6.2)	3083 (8)	4225 (6)
Gestational diabetes	1280 (0.6)	1603 (0.4)	232 (0.6)	253 (0.4)
Pre-eclampsia	812 (0.4)	1081 (0.3)	134 (0.4)	203 (0.3)
Pre-term births	1452 (0.7)	2403 (0.6)	228 (0.6)	378 (0.6)
***Baseline cardiovascular diseases***				
Hypertension	2631 (1.2)	3181 (0.8)	471 (1.3)	562 (0.9)
Diabetes	1898 (0.9)	2359 (0.6)	334 (0.9)	392 (0.6)
Ischemic heart disease	119 (0.1)	123 (0.0)	14 (0.0)	22 (0.0)
Stroke/TIA	307 (0.1)	379 (0.1)	50 (0.1)	73 (0.1)
Heart failure	46 (0.0)	76 (0.0)	12 (0.0)	14 (0.0)

*BMI*, body mass index; *IQR*, inter quartile range; *Kg/m*^*2*^, kilograms per meter square. There were no missing data for age. The total number (%) of missing data for Townsend deprivation quintile, BMI, and alcohol status were 114 579 (16.3%), 184,391 (26.2%), and 73,392 (10.4%), respectively. For current lipid medication, connective tissue disorders, migraine, reproductive factors, and baseline cardiovascular diseases absence of a diagnostic code for these conditions was assumed to indicate absence of disease

### Composite CVD

#### Menstrual cycle regularity

**Fig. 1:**
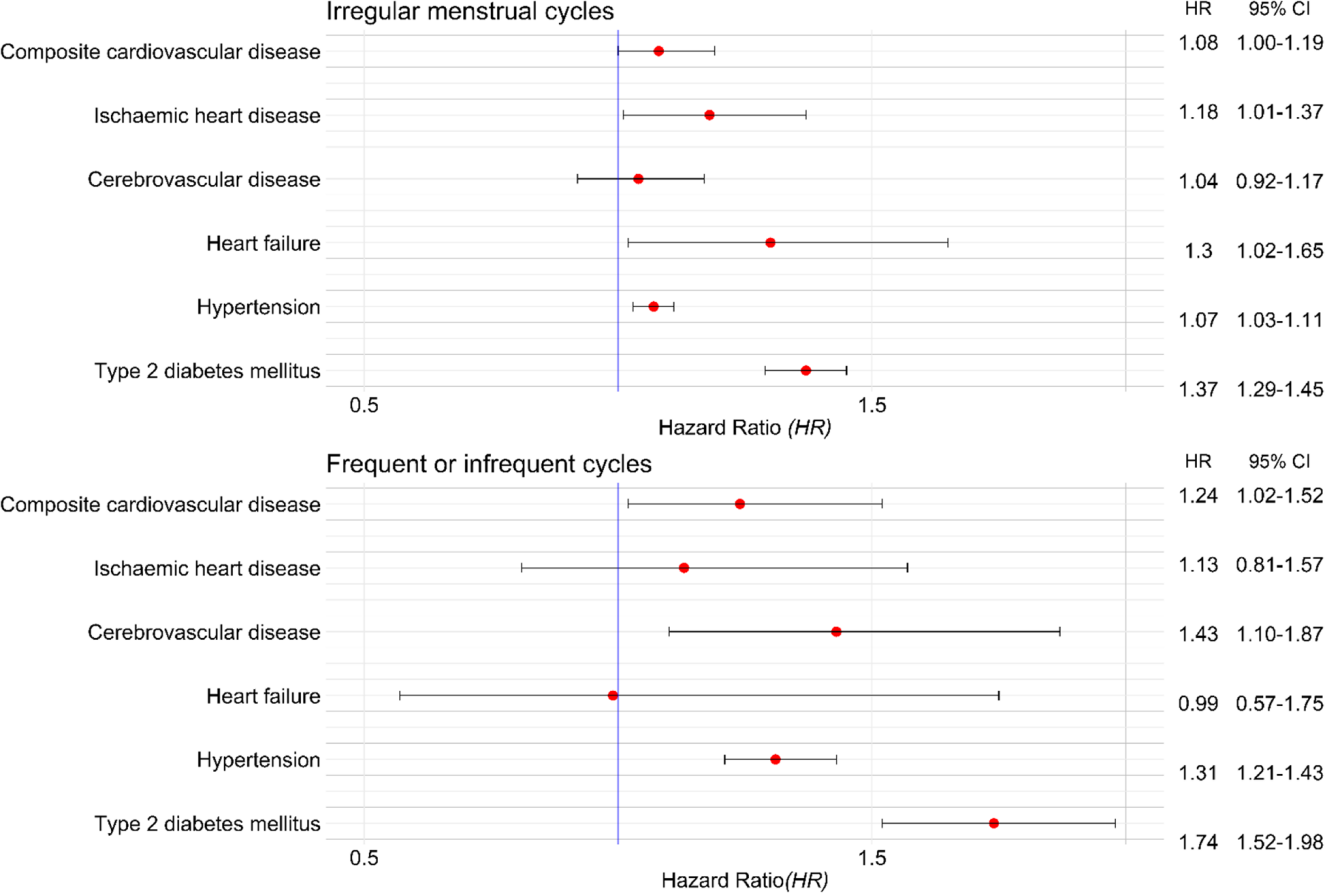
Forest plot showing the fully adjusted effect estimates and 95% CI for cardiometabolic outcomes in women with history of irregular menstrual cycles or frequent or infrequent menstrual cycles

**Fig. 2:**
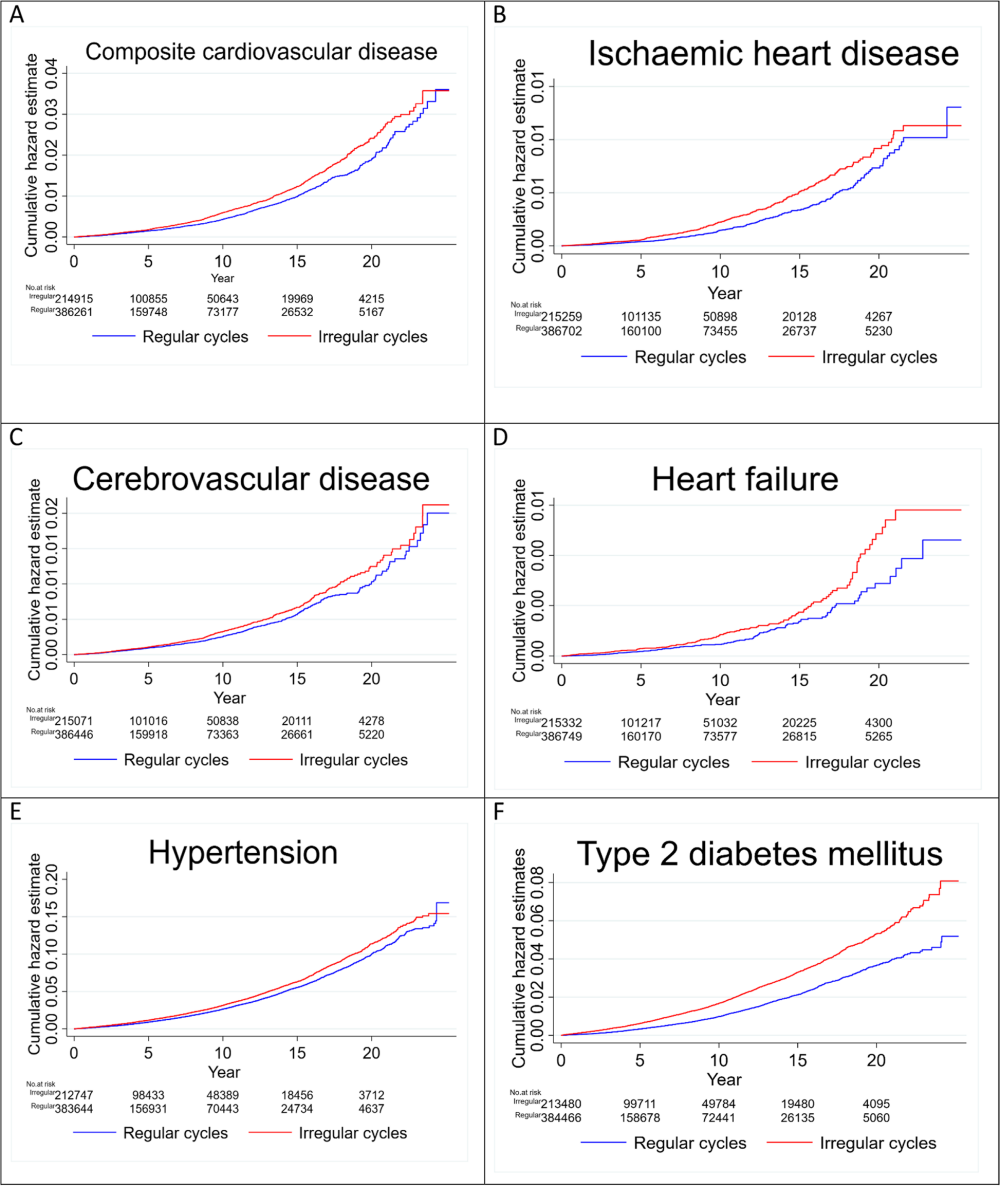
Cumulative hazard estimates of cardiometabolic outcomes (**A-F**) in women with irregular cycles compared to those with regular cycles

Between 1995 and 2021, 896 and 1056 composite CVD events were recorded among women with irregular versus regular menstrual cycles, respectively. Median (IQR) follow-up was 4.5 (1.7–9.6) years in the exposed and 3.8 (1.4–8.3) years in the unexposed group. The crude incidence rate (per 1000 years) of composite CVD was 0.67 in women with irregular menstrual cycles versus 0.50 in women with regular menstrual cycles. The HR for composite CVD comparing irregular with regular menstrual cycles were 1.26 (95% CI 1.15–1.38; *p* < 0.001) in the crude model and 1.08 (95% CI 1.00–1.19; *p* = 0.062) in the model adjusting for sociodemographic, lifestyle, medical, and reproductive characteristics (Figs. 1, 2, Additional file: Table S3).

#### Menstrual cycle frequency

**Fig. 3:**
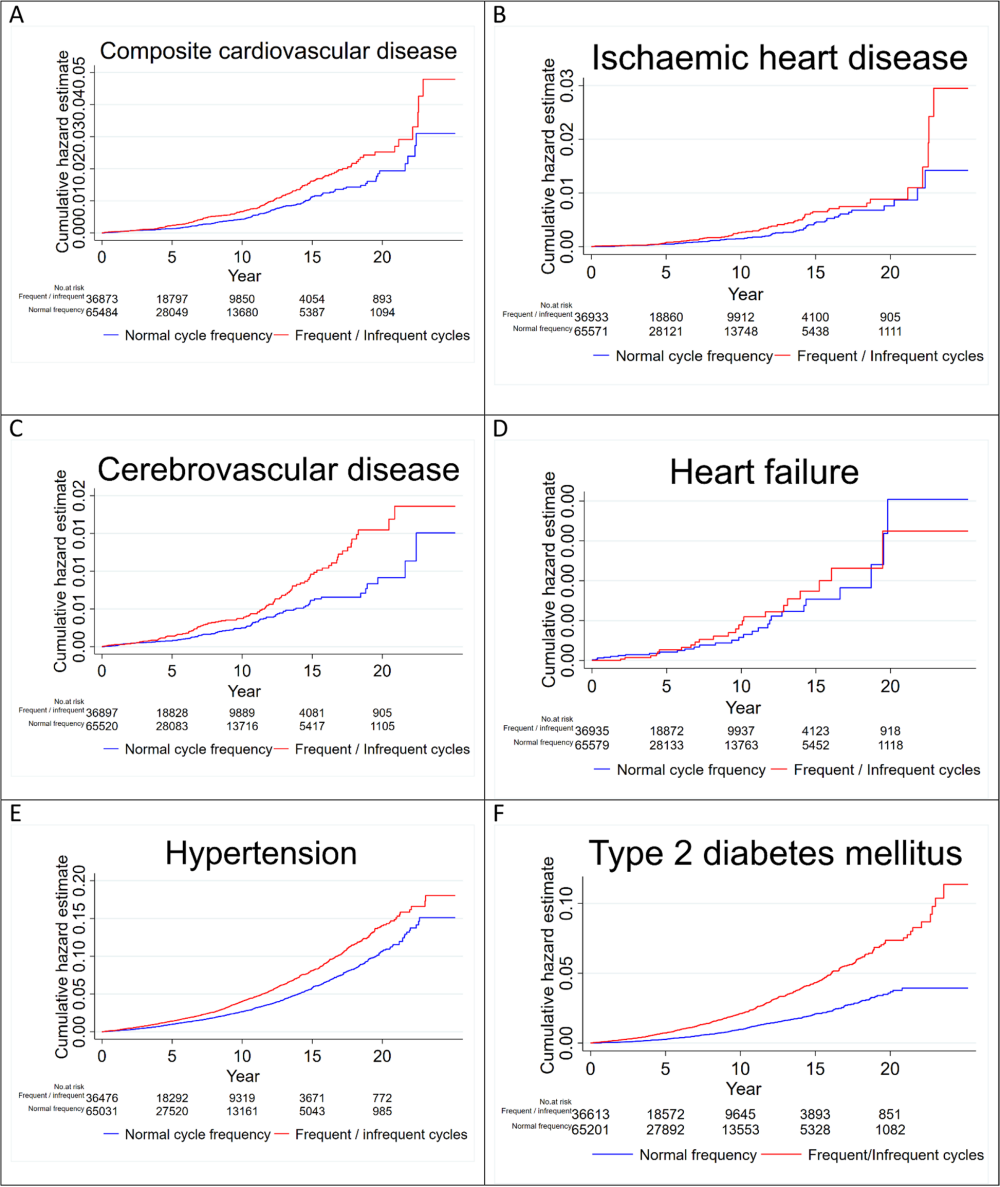
Cumulative hazard estimates of cardiometabolic outcomes (**A-F**) in women with frequent or infrequent cycles compared to those with normal cycle frequency

During the study period, 205 versus 202 composite CVD events were recorded in women with frequent or infrequent menstrual cycles compared to controls with menstrual cycles of normal frequency, respectively. Median (IQR) follow-up was 5.1 (2.0–10.5) years in the exposed and 4.0 (1.5–8.8) years in the unexposed group. The crude incidence rate (per 1000 person years) of composite CVD was 0.83 in women with frequent or infrequent cycles compared to 0.53 in women with menstrual cycles of normal frequency with a crude HR of 1.46 (95% CI 1.20–1.78; *p* < 0.001) (Additional file: Table S3). In the adjusted model, the association between frequent or infrequent cycles and composite CVD was maintained (aHR 1.24, 95% CI 1.02–1.52; *p* = 0.031) (Figs. 1, 3).

### CVD subtypes

#### Menstrual cycle regularity

In the model comparing irregular to regular menstrual cycles, the adjusted HR for CVD subtypes were as follows: 1.18 (95% CI 1.01–1.37; *p* = 0.033) for ischemic heart disease, 1.04 (95% CI 0.92–1.17; *p* = 0.508) for cerebrovascular disease, and 1.30 (95% CI 1.02–1.65; *p* = 0.033) for heart failure (Figs. 1, 2, Additional file: Table S3).

#### Menstrual cycle frequency

In the model comparing frequent or infrequent menstrual cycles to menstrual cycles of normal frequency, the adjusted HR for CVD subtypes were 1.13 (95% CI 0.81–1.57; *p* = 0.464) for ischemic heart disease, 1.43 (95% CI 1.10–1.87; *p* = 0.007) for cerebrovascular disease, and 0.99 (95% CI 0.57–1.75; *p* = 0.985) for heart failure (Figs. 1, 2, Additional file: Table S3).

### Hypertension

#### Menstrual cycle regularity

During follow-up, the crude incidence rate (per 1000 person-years) of hypertension was 3.48 in women with irregular menstrual cycles versus 2.79 in controls with regular menstrual cycles. Compared to those with regular menstrual cycles, women with irregular menstrual cycles had a HR of subsequent hypertension of 1.19 (95% CI 1.14–1.24; *p* < 0.001) and 1.07 (95% CI 1.03–1.11; *p* = 0.001) in the unadjusted and adjusted models, respectively (Figs. 1, 2, and Additional file: Table S3).

#### Menstrual cycle frequency

The crude incidence rate (per 1000 person-years) of hypertension was 4.42 in women with frequent or infrequent menstrual cycles compared to 3.0 in women with menstrual cycles of normal frequency, with a crude HR of 1.41 (95% CI 1.30–1.54; *p* < 0.001). In the adjusted model, women with frequent or infrequent cycles were 32% more likely to develop hypertension (HR 1.31; 95% CI 1.21–1.43; *p* < 0.001) (Figs. 1, 3, and Additional file Table S3).

### Type 2 diabetes mellitus

#### Menstrual cycle regularity

The crude incidence rate (per 1000 person-years) of type 2 diabetes mellitus was 1.82 in women with irregular menstrual cycles and 1.05 in those with regular menstrual cycles. Compared to women with regular menstrual cycles, women with irregular menstrual cycles were more likely to develop type 2 diabetes mellitus in both the crude (HR 1.66; 95% CI 1.34–1.49; *p* < 0.001) and adjusted (1.37; 95% CI 1.29–1.45; *p* < 0.001) models (Figs. 1, 2, and Additional file Table S3).

#### Menstrual cycle frequency

The crude incidence rate (per 1000 years) of type 2 diabetes mellitus was 2.38 in women with frequent or infrequent menstrual cycles versus 1.02 in women with menstrual cycles of normal frequency. Women with frequent or infrequent cycles were twice as likely to develop type 2 diabetes mellitus compared to women with menstrual cycles of normal frequency (crude HR 2.25; 95% CI 1.96–2.53; *p* < 0.001). The association was maintained in the adjusted model (HR 1.74; 95% CI 1.52–1.98; *p* < 0.001) (Figs. 1, 3, Additional file: Table S3).

### Sensitivity analyses

#### Menstrual cycle regularity

The effect estimate for the association between menstrual cycle irregularity and composite CVD showed only minimal changes on exclusion of women with amenorrhea (aHR 1.09; 95% CI 0.96–1.24; *p* = 0.173), polycystic ovary syndrome (aHR 1.09; 95% CI 0.99–1.19; *p* = 0.080), endometriosis (aHR 1.09; 95% CI 0.99–1.20; *p* = 0.068), fibroids (aHR 1.09; 95% CI, 0.99–1.19; *p* = 0.067), or current oral contraceptive use (aHR 1.03; 95% CI 0.94–1.15; *p* = 0.445) (Additional file: Table S4). Also, the effect estimate for the association between menstrual cycle irregularity and composite CVD showed only minimal changes (aHR 1.09 (95% CI, 1.00–1.20; *p* = 0.052) on excluding polycystic ovary syndrome, endometriosis, and fibroids as covariates included in the multivariable Cox proportional hazard model (Additional file: Table S5).

#### Menstrual cycle frequency

The association between frequent or infrequent cycles and composite CVD was no longer maintained on exclusion of women with amenorrhea (aHR 1.18; 95% CI 0.95–1.47; *p* = 0.130) and on current oral contraceptive use (aHR 1.14; 95% CI 0.91–1.42; *p* = 0.259). The association between frequent or infrequent cycles and risk of composite CVD was sustained on exclusion of women with history of polycystic ovary syndrome (aHR 1.23; 95% CI 1.01–1.51; *p* = 0.043), endometriosis (aHR 1.24; 95% CI 1.02–1.52; *p* = 0.035), or uterine fibroids (aHR 1.26; 95% CI 1.03–1.54; *p* = 0.025) (Additional file: Table S6). The effect estimate for composite CVD for the association between frequent or infrequent cycles and composite CVD was not materially affected (aHR 1.28 95% CI, 1.05–1.55 *p* = 0.016)) on exclusion of polycystic ovary syndrome, endometriosis, and fibroids as covariates included in adjusted Cox proportional hazard model (Additional file: Table S5). In the analysis comparing frequent menstrual cycles to menstrual cycles of normal frequency, the adjusted HRs for cardiometabolic outcomes were as follows: 1.42 (95% CI 1.09–1.85; *p* = 0.009) for composite CVD, 1.13 (95% CI 0.74–1.72; *p* = 0.570) for IHD, 1.88 (95% CI 1.33–2.67; *p* < 0.001) for cerebrovascular disease, 0.93 (95% CI, 0.42–2.06; *p* = 0.858) for heart failure, 1.37 (95% CI 1.22–1.54; *p* < 0.001) for hypertension, and 1.37 (95% CI 1.13–1.65; *p* < 0.001) for type 2 diabetes mellitus (Additional file: Table S7). For the analysis examining infrequent menstrual cycles versus menstrual cycles of normal frequency, the adjusted HRs for cardiometabolic outcomes were 1.06 (95% CI 0.78–1.45; *p* = 0.704) for composite CVD, 1.16 (95% CI 0.68–1.97; *p* = 0.582) for IHD, 1.01 (95% CI 0.66–1.53; *p* = 0.980) for cerebrovascular disease, 1.13 (95% CI 0.50–2.54; *p* = 0.770) for heart failure, and 1.24 (95% CI 1.85–2.72; *p* < 0.001) for type 2 diabetes mellitus (Additional file: Table S7).

There was no evidence of interaction between cycle dysfunction (irregular and frequent or frequent) and lifestyle characteristics including BMI, smoking, and alcohol consumption (Additional file: Figure S2).

## Discussion

### Main findings

In this nationwide cohort study of more than 700 thousand women from the UK, history of both irregular menstrual cycles and frequent or infrequent menstrual cycles were associated with an increased risk of several cardiometabolic outcomes. The associations were strongest for women with abnormal patterns in the frequency of their menstrual cycles with frequent or infrequent cycles being associated with a significant increase in hazard of composite CVD. History of menstrual cycle irregularity was associated with a borderline increase in the hazard of composite CVD. On examination by subtypes of CVD, menstrual cycle irregularity was associated with an increased risk of ischemic heart disease and heart failure but not cerebrovascular disease. Frequent or infrequent cycles were associated with an increased risk of cerebrovascular disease but not ischemic heart disease or heart failure. On examination by subtype of menstrual cycle frequency, frequent menstrual cycles were associated with an elevated risk of composite CVD and cerebrovascular disease but not ischemic heart disease or heart failure. No association was observed between infrequent menstrual cycles and composite CVD or any of the CVD subtypes. Both irregular menstrual cycles and frequent or infrequent cycles were linked with an increased risk of hypertension and type 2 diabetes mellitus.

### Comparison with previous literature

A summary of the study characteristics of selected existing literature are provided in Additional file: Table S8 [8, 9, 34–40]. Overall, results from our study support and expand existing literature that have examined the association between menstrual characteristics and cardiometabolic outcomes. A UK prospective cohort study of 40,896 premenopausal women aged 50 years and below at baseline examined the association between irregular menstrual cycles and risk of fatal and non-fatal cardiovascular disease [34]. During a median duration of follow-up of 6.9 years (IQR: 6.2 to 7.6), no relationship was found between irregular menstrual cycles compared to regular menstrual cycles and risk of fatal and non-fatal CVD outcomes (Additional file: Table S8). Three United States (US) prospective cohort studies that were all conducted by Wang et al. examined the relationship between menstrual cycle characteristics and cardiometabolic outcomes (CVD and diabetes mellitus) in a cohort of female nurses [8, 9, 35]. The studies by Wang et al. typically defined menstrual cycle regularity as very regular, regular, usually irregular, and always irregular or no period, while cycle length was defined as ≤ 25 days, 26–31 days, 32–39 days, and ≥ 40 days. The most recent study by Wang et al. [9] followed up 80,630 women for a period of 24 years to examine relationship between menstrual cycle regularity and risk of CVD (fatal and non-fatal). Compared to women who had very regular cycles at ages 14 to 17 years, 18 to 22 years, and 29 to 46 years, women with always irregular cycles or no periods at ages 18 to 22 and 29–46 age groups were at an elevated risk of CVD in later life (Additional file: Table S8). In the second prospective cohort study [8], Wang and colleagues followed up 79,505 premenopausal women for a period of 24 years to evaluate the association between menstrual cycle characteristics and risk of premature mortality. Always irregular cycle or no period at ages 18–46 was associated with mortality from CVD (Additional file: Table S8.) [35]. Wang et al. followed 75,456 participants followed for a period of 24 years to investigate the association between menstrual cycle characteristics and type 2 diabetes mellitus. Both irregular menstrual cycles and menstrual cycle length of ≥ 40 days were associated with an elevated risk of type 2 diabetes mellitus (Additional file: Table S8). A recent Australian study of 13,714 participants investigated the relationship between irregular menstrual cycles compared to regular menstrual cycles (never, sometimes, or rarely) and risk of non-fatal heart disease (myocardial infarction, angina) [36]. During the 20-year period of follow-up, irregular menstrual cycles compared to regular menstrual cycles were linked to higher risk of heart disease and diabetes mellitus (Additional file: Table S8). We observed a relationship between frequent (short) but not infrequent (long) menstrual cycles and CVD. This contrasted with a US cohort study which found an association between long menstrual cycles but not short menstrual cycles and CVD [9]. In the US study, compared to women with a menstrual cycle length of 26–31 days, the adjusted HRs for cardiovascular disease were as follows: 1.00 (95% CI 0.83–1.20) for cycle lengths of ≤ 25 days, 1.05 (95% CI 0.89–1.24) for cycle lengths of 32–39 days, and 1.30 (95% CI 1.09–1.57) for cycle lengths of ≥ 40 days or too irregular to estimate (Additional file: Table S8) [9]. In our study, menstrual cycle frequency was classified as either frequent or infrequent. Our study could not differentiate menstrual cycle frequency by cycle length in days. Therefore, our results should be interpreted with caution given that the relationship between infrequent (long) menstrual cycles and CVD appears to be greatest with increasing length (≥ 40 days) in menstrual cycles, as suggested by the findings from the US study [9]. The association between frequent (short) menstrual cycles and elevated CVD risk is biologically plausible. Frequent menstrual cycles are a marker of diminished ovarian reserve [41]. Previous studies have reported a relationship between diminished ovarian reserve and elevated CVD risk [42, 43].

An Iranian study followed up 2128 women aged 18–49 years at baseline to investigate the association between irregular menstrual cycles compared regular menstrual cycles and risk of cardiometabolic outcomes [37]. During the 15-year period of follow-up, irregular menstrual cycles compared to regular menstrual cycles were associated with higher risk of type 2 diabetes mellitus but not hypertension. The present study found that irregular menstrual cycles were associated with an increased risk of heart failure. However, due to the low number of events, we did not find any association between changes in menstrual cycle frequency and heart failure risk. Direct comparisons between the existing literature and the present study are challenging due to several differences which may partly explain some of the contrasting findings. The main methodological differences relate to the stratification of irregular menstrual cycles by severity into four categories (US studies) [8, 9, 35]: case definition of the exposure to include both regularity and frequency of menses as a single exposure (Iranian study) [37]; case definition of the unexposed group (regular menstrual cycles) as never, rarely, or sometimes (Australian study) [36]; restriction of the study participants exclusively to nurses (US studies) [8, 9, 35]; and inclusion of fatal CVD events in the outcomes (US and UK studies) [9, 34].

### Biological plausibility

Several mechanisms yet to be fully elucidated are suspected to play a role in the association between menstrual cycle characteristics and elevated risk of cardiometabolic outcomes. First, PCOS which a common cause of amenorrhea, irregular menstrual cycles, and oligomenorrhea is characterized by cardiovascular risk factors including metabolic syndrome, obesity, insulin resistance, dyslipidemia, and hypertension [44]. The present study found that the association between menstrual complications and cardiometabolic outcomes was independent of PCOS. That PCOS is associated with an increased risk of CVD is debatable. Some studies report an increased risk of CVD among women with PCOS, while other studies argue that any observed association is minimal or restricted to severe phenotypes of PCOS [45]. Second, other reproductive factors (endometriosis, fibroids) associated with changes in menstrual characteristics and linked to adverse cardiometabolic health may partly account for the observed association [46, 47]. However, exclusion of women with a record for endometriosis or fibroids in sensitivity analyses did not alter the observed effect estimates. Attenuation of the effect size on exclusion of women on current prescription for combined oral contraceptive (COC) suggests that increased CVD risk may be partly mediated by COC use [48]. Third, changes in menstrual cycle characteristics are strongly linked to hyperinsulinemia. Hyperinsulinemia suppresses the production of sex hormone-binding globulin resulting in elevated level of free testosterone. This hormonal environment is associated with higher risk of cardiometabolic outcomes [49–52]. Fourth, estrogen modulates vascular inflammation [53, 54]. Abnormal menstrual patterns may favor pro-inflammatory process which may result in atherosclerotic CVD. Fifth, differences in mechanistic pathways between menstrual cycle characteristics may partly account for the differences in findings. A longer cycle length may be indicative of fewer ovulations and, consequently, lower mean estrogen levels [39]. Higher levels of endogenous estradiol before menopause have been associated with a decreased risk of subclinical atherosclerosis after menopause [55]. Short menstrual cycle length may be an indicator of ovarian aging [41, 56]. Markers of diminished ovarian reserve including anti-Müllerian hormone (AMH) and elevated follicle stimulating hormone (FSH) have been associated with CVD risk factors [57, 58]. In addition, low AMH levels may act independently to promote atherogenesis [42, 59].

### Strengths and limitations

The main strength of the present study is the use of a large sample size that is representative of the UK population and a long duration of follow-up that allows sufficient time for the development of cardiometabolic outcomes. Unlike previous studies that relied on self-reports of the exposure several years after their occurrence, the present study relied on electronic health data documented at point of clinical consultation which helped to minimize recall bias. In addition, we adjusted for several key sociodemographic, lifestyle, medical, and reproductive characteristics. Several limitations should be acknowledged. Foremost, we could not characterize menstrual cycle characteristics by grades of severity or duration as this information was not coded in UK electronic health records. Second, the exposure of interest relies on self-report and is therefore susceptible to misclassification. Third, although we adjusted for several known and potential confounders, the possibility of unmeasured confounding remains; for instance, we were not able to adjust for dietary habits, physical activity, or family history of CVD as this information is not well recorded in UK primary care data. Fourth, where a patient had a diagnostic code for both irregularity and frequent or infrequent cycles, exposure status was assigned as the first ever recorded menstrual cycle characteristic domain. This makes the implicit assumption that the order in which these conditions are recorded is random; however, this may not be the case. Nevertheless, given that participants impacted by any potential classification bias will have had both menstrual cycle characteristics (and could therefore contribute to either exposure definition), and the direction of effect for most outcomes was similar for the two exposures, we expect this to have a limited impact on the findings. Fifth, there is potential for exposure misclassification among women who were included in the unexposed cohort but had abnormal menstrual cycle characteristics not recorded in primary care or who were on hormonal contraceptives. Also, we did not exclude women with abnormal cycle characteristic shortly after pregnancy or during lactation. Although history of breastfeeding compared to no breastfeeding is associated with reduced maternal risk CVD, hypertension, and diabetes mellitus [60, 61], we were not able to adjust for history of breastfeeding in the analysis. Sixth, a further drawback is the possibility for selection bias due to differential loss to follow-up between exposure groups: 40.7% of women in the exposed groups and 46.0% in the unexposed groups were lost to follow-up due to leaving the general practice.

### Implications for public health and research

Findings from the present study support calls for the inclusion of menstrual cycle history as an additional vital sign in the assessment of the overall health status of young women. Specifically, abnormal menstruation may act as a window into the future cardiometabolic health of women. Therefore, women with history of irregular menstrual cycles or frequent or infrequent menstrual cycles may benefit from periodic evaluation of their cardiometabolic health. Current UK guidelines should consider incorporating reproductive factors including menstrual cycle characteristics as risk enhancing factors for cardiometabolic disease given the low awareness about these factors among UK physicians [62]. Future research should determine the pathophysiological mechanisms linking menstrual cycle complications and adverse cardiometabolic health and the factors behind the differential impact of different menstrual cycle characteristics and poor cardiometabolic outcomes.

## Conclusions

History of irregular menstrual cycles or frequent or infrequent menstrual cycles is associated with increased risk of cardiometabolic outcomes in later-life. Research is needed to unravel the pathophysiological links behind changes in menstrual cycle and adverse cardiometabolic health. Incorporating reproductive history including menstrual cycle characteristics as part of routine medical evaluation may help identify potential candidates for periodic assessment of cardiometabolic health.

## Supplementary Information

**Additional file1: Tables S1-S8** and **Figures S1-S2.** **Table S1.** Classification of menstrual cycle characteristics. **Table S2.** Diagnostic Read codes for menstrual cycle regularity and menstrual cycle frequency. **Table S3.** Incidence rates and hazard ratios of cardiometabolic outcomes. **Table S4.** Sensitivity analyses menstrual cycle regularity and composite CVD analyses (excluding women with amenorrhea, polycystic ovary syndrome, endometriosis, current hormonal contraceptive use, uterine fibroids). **Table S5.** Incidence rates and hazard ratio for cardiometabolic outcomes (sensitivity analyses excluding polycystic ovarian syndrome, endometriosis, and fibroids as covariates from adjusted Cox proportional hazard model). **Table S6.** Sensitivity analyses menstrual cycle frequency and composite CVD (excluding women with amenorrhea, polycystic ovary syndrome, endometriosis, current hormonal contraceptive use, uterine fibroids). **Table S7.** Incidence rates and hazard ratios for cardiometabolic outcomes among women with frequent (short) menstrual cycles and infrequent (Long) menstrual cycle. **Table S8**. Summary of selected existing literature examining the association between menstrual characteristics and cardiometabolic outcomes. **Figure S1.** Study participant flow chart. **Figure S2.** Interaction between (A) irregular menstrual cycles and (B) frequent or infrequent menstrual cycles and lifestyle factors (Body mass index, smoking, and alcohol use).

## Data Availability

The data that support the findings of this study are available from IQVIA but restrictions apply to the availability of these data, which were used under license for the current study, and so are not publicly available. Data are however available from the authors upon reasonable request and with permission of IQVIA**.**

## Abbreviations

aHR: Adjusted hazard ratio
BMI: Body mass index
COC: Combined oral contraceptive
CVD: Cardiovascular disease
HF: Heart failure
IHD: Ischemic heart disease
IMRD: IQVIA Medical Research Data
IQR: Interquartile range
PCOS: Polycystic ovary syndrome
QOF: Quality and Outcomes Framework
T2DM: Type 2 diabetes mellitus
THIN: The Health Improvement Network
UK: United Kingdom
US: United States of America
WHO: World Health Organization
