# Supplementary material for: The association between menstrual cycle characteristics and cardiometabolic outcomes in later life: a retrospective matched cohort study of 704,743 women from the UK
Source: BMC Med. 2023 Mar 20;21:104. doi: 10.1186/s12916-023-02794-x (PMC10029324; doi:10.1186/s12916-023-02794-x)
Supplement: Supplementary file 1 — Additional file1: Tables S1-S8 and Figures S1-S2. Table S1. Classification of menstrual cycle characteristics. Table S2. Diagnostic Read codes for menstrual cycle regularity and menstrual cycle frequency. Table S3. Incidence rates and hazard ratios of cardiometabolic outcomes. Table S4. Sensitivity analyses menstrual cycle regularity and composite CVD analyses (excluding women with amenorrhea, polycystic ovary syndrome, endometriosis, current hormonal contraceptive use, uterine fibroids). Table S5. Incidence rates and hazard ratio for cardiometabolic outcomes (sensitivity analyses excluding polycystic ovarian syndrome, endometriosis, and fibroids as covariates from adjusted Cox proportional hazard model). Table S6. Sensitivity analyses menstrual cycle frequency and composite CVD (excluding women with amenorrhea, polycystic ovary syndrome, endometriosis, current hormonal contraceptive use, uterine fibroids). Table S7. Incidence rates and hazard ratios for cardiometabolic outcomes among women with frequent (short) menstrual cycles and infrequent (Long) menstrual cycle. Table S8. Summary of selected existing literature examining the association between menstrual characteristics and cardiometabolic outcomes. Figure S1. Study participant flow chart. Figure S2. Interaction between (A) irregular menstrual cycles and (B) frequent or infrequent menstrual cycles and lifestyle factors (Body mass index, smoking, and alcohol use). [file 12916_2023_2794_MOESM1_ESM.docx]

**Additional file 1**

**Tables**

**Table S1:** Classification of menstrual cycle characteristics

**Table S2**: Diagnostic Read codes for menstrual cycle regularity and menstrual cycle frequency.

**Table S3:** Incidence rates and hazard ratios of cardiometabolic outcomes

**Table S4:** Sensitivity analyses menstrual cycle regularity and composite CVD analyses (excluding women with amenorrhoea, polycystic ovary syndrome, endometriosis, current hormonal contraceptive use, uterine fibroids)

**Table S5:** Incidence rates and hazard ratio for cardiometabolic outcomes (sensitivity analyses excluding polycystic ovarian syndrome, endometriosis, and fibroids as covariates from adjusted Cox proportional hazard model)

**Table S6**: Sensitivity analyses menstrual cycle frequency and composite CVD (excluding women with amenorrhoea, polycystic ovary syndrome, endometriosis, current hormonal contraceptive use, uterine fibroids)

**Table S7**: Incidence rates and hazard ratios for cardiometabolic outcomes among women with frequent (short) menstrual cycles and infrequent (Long) menstrual cycle.

**Table S8:** Summary of selected existing literature examining the association between menstrual characteristics and cardiometabolic outcomes.

**Figures**

**Figure S1**: Study participant flow chart.

**Figure S2**: Interaction between (A) irregular menstrual cycles and (B) frequent or infrequent menstrual cycles and lifestyle factors (Body mass index, smoking, and alcohol use)

**Table S1**: **Classification of menstrual characteristics.**

| **Menstrual characteristic** | **Description** | **Normal limits (5^th^ to 95^th^ percentile)** |
| --- | --- | --- |
| Frequency of menses | Frequent | < 24 days |
|  | Normal | 24-38 days |
|  | Infrequent | >38 days |
| Regularity of menses (cycle to cycle variation over 12 months) | Absent | No |
|  | Regular | Variation +2 to 20 days |
|  | Irregular | Variation greater than 20 days |
| Duration of flow | Prolonged | >8.0 days |
|  | Normal | 4.5—8.0 days |
|  | Shortened | < 4.5 days |
| Volume of blood loss per months | Heavy | >80 millilitres |
|  | Normal | 5—80 millilitres |
|  | Light | 5 < millilitres |

**Table S2: Read codes for cycle irregularity and cycle frequency.**

The relevant diagnostic and other health related code lists were generated through a careful process that involves the following sequential steps; (i) Development of a comprehensive list of search terms, (ii) A search of the Read code dictionary for relevant codes using the list of search terms, (iii) A search of additional relevant codes lists from online Read code repositories, or supplemental information of published studies, (iv) Rating each code in the code list for relevance and deciding on the final list by consulting clinical experts (general practitioners and consultant specialists)

| READ CODE | DESCRIPTION | Classification |
| --- | --- | --- |
| 1571 | H/O: amenorrhoea | Regularity of menses |
| K590.00 | Absence of menstruation | Regularity of menses |
| K590.11 | Amenorrhoea | Regularity of menses |
| K590000 | Primary amenorrhoea | Regularity of menses |
| K590z00 | Amenorrhoea NOS | Regularity of menses |
| K594.00 | Irregular menstrual cycle | Regularity of menses |
| K594z00 | Irregular menstrual cycle NOS | Regularity of menses |
| K598.00 | Menometrorrhagia | Regularity of menses |
| K59y.11 | Metropathia haemorrhagica | Regularity of menses |
| Kyu9C00 | [X]Other specified irregular menstruation | Regularity of menses |
| 1572 | H/O: polymenorrhoea | Frequency of menses |
| K591.00 | Scanty or infrequent menstruation | Frequency of menses |
| K591.11 | Infrequent menstruation | Frequency of menses |
| K591100 | Oligomenorrhoea | Frequency of menses |
| K591200 | Primary oligomenorrhoea | Frequency of menses |
| K591z00 | Scanty or infrequent menstruation NOS | Frequency of menses |
| K592.00 | Excessive or frequent menstruation | Frequency of menses |
| K592.11 | Frequent menses | Frequency of menses |
| K592100 | Polymenorrhoea | Frequency of menses |
| K592111 | Epimenorrhoea | Frequency of menses |
| K592z00 | Excessive or frequent menstruation NOS | Frequency of menses |

| 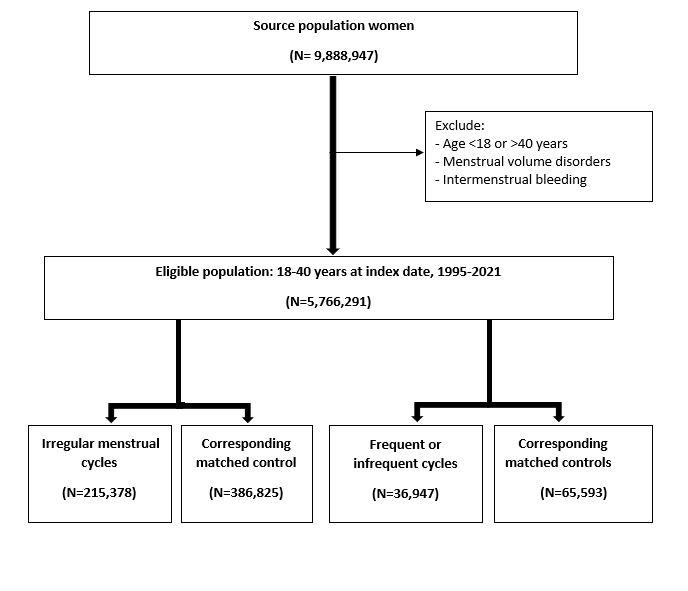 |
| --- |

Figure S1: Study participant flow chart.

**Table S3: Incidence rates and hazard ratio for cardiometabolic outcomes**

|  | **Menstrual cycle regularity** | | **Menstrual cycle frequency** | |
| --- | --- | --- | --- | --- |
|  | **Irregular (exposed)** | **Regular (Unexposed)** | **Frequent/Infrequent (exposed)** | **Normal (unexposed)** |
| **Composite CVD** |  |  |  |  |
| Population | 214,915 | 386,261 | 36,873 | 65,484 |
| Events, n (%) | 896 (0.42%) | 1056 (0.27%) | 205 (0.56%) | 202 (0.31%) |
| Person-years | 1339541 | 2121954 | 248301.2 | 377644.7 |
| Crude incident rate/1000 years | 0.67 | 0.50 | 0.83 | 0.53 |
| Age at outcome Median (IQR) | 43.1 (37.4-47.8) | 42.7 (37.6-47.8) | 43.2 (38.5 – 48.5) | 43.5 (37.4 -48.2) |
| Crude HR (95% CI) | 1.26 (1.15-1.38) |  | 1.46 (1.20-1.78) |  |
| P-value | < 0.001 |  | <0.001 |  |
| Adjusted HR (95% CI) | 1.08 (1.00-1.19) |  | 1.24 (1.02-1.52) |  |
| P-value | 0.062 |  | 0.031 |  |
| **IHD** |  |  |  |  |
| Population | 215,259 | 386,702 | 36,933 | 65,571 |
| Events, n (%) | 342 (0.16%) | 353 (0.09%) | 77 (0.21%) | 76 (0.12%) |
| Person-years | 1344052 | 2127601 | 249369.3 | 378907.9 |
| Crude incident rate/1000 years | 0.24 | 0.17 | 0.30 | 0.20 |
| Age at outcome Median (IQR) | 44.0 (40.1-48.4) | 44.6 (40.3-49.8) | 45.1 (40.5-49.2) | 45.8 (40.9-51.0) |
| Crude HR (95% CI) | 1.43 (1.23-1.66) |  | 1.44 (1.05-1.97) |  |
| P-value | < 0.001 |  | 0.025 |  |
| Adjusted HR (95% CI) | 1.18 (1.01-1.37) |  | 1.13 (0.81-1.57) |  |
| P-value | 0.033 |  | 0.464 |  |
| **Cerebrovascular disease** |  |  |  |  |
| Population | 215,071 | 386,446 | 36,897 | 65,520 |
| Events, n (%) | 494 (0.22%) | 620 (0.16%) | 124 (0.34%) | 111 (0.17%) |
| Person-years | 1342726 | 2125102 | 248888 | 378288 |
| Crude incident rate/1000 years | 0.37 | 0.29 | 0.50 | 0.29 |
| Age at outcome Median (IQR) | 42.5 (35.8-47.2) | 42 (36.1-47.3) | 43.2 (38.5-48.6) | 42.7 (35.3-47.6) |
| Crude HR (95% CI) | 1.19 (1.06- 1.34) |  | 1.62(1.26-2.00) |  |
| P-value | 0.004 |  | < 0.001 |  |
| Adjusted HR (95% CI) | 1.04 (0.92-1.17) |  | 1.43 (1.10-1.87) |  |
| P-value | 0.508 |  | 0.007 |  |
| **Heart failure** |  |  |  |  |
| Population | 215,332 | 386,749 | 36,935 | 65,579 |
| Events, n (%) | 139 (0.06%) | 138 (0.04%) | 23 (0.06%) | 30 (0.05%) |
| Person-years | 1346027 | 2129232 | 249715 | 379180 |
| Crude incident rate/1000 years | 0.1 | 0.06 | 0.09 | 0.08 |
| Age at outcome Median (IQR) | 44.4 (37.9 - 49) | 41.5 (36.7-46.9) | 45.2 (35.4-48.9) | 39.9 (33.6-45.7) |
| Crude HR (95% CI) | 1.48 (1.17-1.87) |  | 1.10 (0.64-1.90) |  |
| P-value | 0.001 |  | 0.723 |  |
| Adjusted HR (95% CI) | 1.30 (1.02-1.65) |  | 0.99 (0.57-1.75) |  |
| P-value | 0.033 |  | 0.985 |  |
| **Hypertension** |  |  |  |  |
| Population | 212,747 | 383,644 | 36,476 | 65,031 |
| Events, n (%) | 4529 (2.13%) | 5788 (1.51%) | 1060 (2.91%) | 1109 (1.71%) |
| Person-years | 1300272 | 2076051 | 239680 | 369126 |
| Crude incident rate/1000 years | 3.48 | 2.79 | 4.42 | 3.00 |
| Age at outcome Median (IQR) | 41.6 (36.4-46.2) | 41.8 (36.8-46.6) | 42 (37.3 - 47) | 43 (37.7- 47.8) |
| Crude HR (95% CI) | 1.19 (1.14 - 1.24) |  | 1.41 (1.30-1.54) |  |
| P-value | < 0.001 |  | <0.001 |  |
| Adjusted HR (95% CI) | 1.07 (1.03-1.11)* |  | 1.31 (1.21-1.43) * |  |
| P-value | 0.001 |  | <0.001 |  |
| **Diabetes mellitus** |  |  |  |  |
| Population | 213,480 | 384,466 | 36,613 | 65,201 |
| Events, n (%) | 2412 (1.13%) | 2215 (0.58%) | 582 (1.59%) | 383 (0.59%) |
| Person-years | 1322940 | 2106721 | 244548.3 | 375211.8 |
| Crude incident rate/1000 years | 1.82 | 1.05 | 2.38 | 1.02 |
| Age at outcome Median (IQR) | 40.6 (34.8-45.3) | 41.3 (36.1-46.2) | 40.5 (34.3-46.2) | 42.8 (38.3-47.1) |
| Crude HR (95% CI) | 1.66 (1.56-1.75) |  | 2.25(1.96-2.53) |  |
| P-value | < 0.001 |  | <0.001 |  |
| Adjusted HR (95% CI) | 1.37 (1.29-1.45) # |  | 1.74 (1.52-1.98) # |  |
| P-value | <0.001 |  | <0.001 |  |

Adjusted for age, Townsend quintile of deprivation, body mass index, smoking, lipid medication, Alcohol status, hypertension, diabetes mellitus, current use of combined oral contraceptive, connective tissue disorders, migraine, polycystic ovary syndrome, gestational diabetes mellitus (types 1 and 2), pre-eclampsia, pre-term birth, pelvic inflammatory disease, endometriosis, fibroids.

*= Adjusted for all above except hypertension

#= Adjusted for all above except diabetes mellitus

**Table S4: Incidence rates and hazard ratios for composite CVD (Sensitivity analyses for menstrual cycle regularity)**

| **Composite CVD** | **Amenorrhoea** | | **Polycystic ovary syndrome** | | **Endometriosis** | |
| --- | --- | --- | --- | --- | --- | --- |
|  | **Exposed** | **Unexposed** | **Exposed** | **Unexposed** | **Exposed** | **Unexposed** |
| Population | 87,208 | 386,261 | 202970 | 379839 | 212568 | 382545 |
| Events, n (%) | 304 (0.35%) | 1056 | 847 | 1036 | 879 | 1031 |
| Person-years | 489801 | 2121954 | 1279818 | 2091715 | 1323378 | 2099435 |
| Crude incident rate/1000 years | 0.62 | 0.50 | 0.67 | 0.50 | 0.66 | 0.49 |
| Age at outcome Median (IQR) | 43.2 (38.5-48.5) | 43.5 (37.4-48.2) | 43.3 (37.4-48.0) | 42.7 (37.4-47.8) | 43.1 (37.4-47.9) | 42.7 (37.6-47.8) |
| Crude HR (95% CI) | 1.27 (1.12-1.45) |  | 1.25 (1.14-1.37) |  | 1.27 (1.16-1.39) |  |
| P-value | <0.001 |  | <0.001 |  | <0.001 |  |
| Adjusted HR (95% CI) | 1.09 (0.96-1.24) |  | 1.09 (0.99-1.19) |  | 1.09 (0.99-1.20) |  |
| P-value | 0.173 |  | 0.080 |  | 0.068 |  |

Adjusted for age, Townsend quintile of deprivation, body mass index, smoking, lipid medication, Alcohol status, hypertension, diabetes mellitus, current use of combined oral contraceptive, connective tissue disorders, migraine, polycystic ovary syndrome, gestational diabetes mellitus, pre-eclampsia, pre-term birth, pelvic inflammatory disease, endometriosis, fibroids.

| **Composite CVD** | **Current contraceptive use** | | **Fibroids** | |
| --- | --- | --- | --- | --- |
|  | **Exposed** | **Unexposed** | **Exposed** | **Unexposed** |
| Population | 149170 | 282064 | 214219 | 384998 |
| Events, n (%) | 712 (0.48%) | 889 (0.32%) | 890 (0.42%) | 1047 (0.27%) |
| Person-years | 916037 | 1514532 | 1335370 | 2114492 |
| Crude incident rate/1000 years | 0.78 | 0.58 | 0.67 | 0.50 |
| Age at outcome Median (IQR) | 43.7 (38.1-48.3) | 42.9 (38.1-48.2) | 43.2 (37.4-47.9) | 42.7 (37.6-47.7) |
| Crude HR (95% CI) | 1.24 (1.12-1.36) |  | 1.26 (1.15-1.38) |  |
| P-value | < 0.001 |  | <0.001 |  |
| Adjusted HR (95% CI) | 1.03 (0.94-1.15) |  | 1.09 (0.99-1.19) |  |
| P-value | 0.445 |  | 0.067 |  |

Adjusted for age, Townsend quintile of deprivation, body mass index, smoking, lipid medication, Alcohol status, hypertension, diabetes mellitus, current use of combined oral contraceptive, connective tissue disorders, migraine, polycystic ovary syndrome, gestational diabetes mellitus, pre-eclampsia, pre-term birth, pelvic inflammatory disease, endometriosis, fibroids.

**Table S5: Incidence rates and hazard ratio for cardiometabolic outcomes (sensitivity analyses excluding polycystic ovarian syndrome, endometriosis, and fibroids as covariates from the adjusted model)**

|  | **Menstrual cycle regularity** | | **Menstrual cycle frequency** | |
| --- | --- | --- | --- | --- |
|  | **Irregular (exposed)** | **Regular (Unexposed)** | **Frequent/Infrequent (exposed)** | **Normal (unexposed)** |
| **Composite CVD** |  |  |  |  |
| Population | 214,915 | 386,261 | 36,873 | 65,484 |
| Events, n (%) | 896 (0.42%) | 1056 (0.27%) | 205 (0.56%) | 202 (0.31%) |
| Person-years | 1339541 | 2121954 | 248301.2 | 377644.7 |
| Crude incident rate/1000 years | 0.67 | 0.50 | 0.83 | 0.53 |
| Age at outcome Median (IQR) | 43.1 (37.4-47.8) | 42.7 (37.6-47.8) | 43.2 (38.5 – 48.5) | 43.5 (37.4 -48.2) |
| Crude HR (95% CI) | 1.26 (1.15-1.38) |  | 1.46 (1.20-1.78) |  |
| P-value | < 0.001 |  | <0.001 |  |
| Adjusted HR (95% CI) | 1.09 (1.00-1.20) |  | 1.28 (1.05-1.55) |  |
| P-value | 0.052 |  | 0.016 |  |
| **IHD** |  |  |  |  |
| Population | 215,259 | 386,702 | 36,933 | 65,571 |
| Events, n (%) | 342 (0.16%) | 353 (0.09%) | 77 (0.21%) | 76 (0.12%) |
| Person-years | 1344052 | 2127601 | 249369.3 | 378907.9 |
| Crude incident rate/1000 years | 0.24 | 0.17 | 0.30 | 0.20 |
| Age at outcome Median (IQR) | 44.0 (40.1-48.4) | 44.6 (40.3-49.8) | 45.1 (40.5-49.2) | 45.8 (40.9-51.0) |
| Crude HR (95% CI) | 1.43 (1.23-1.66) |  | 1.44 (1.05-1.97) |  |
| P-value | < 0.001 |  | 0.025 |  |
| Adjusted HR (95% CI) | 1.18 (1.02-1.37) |  | 1.14 (0.82-1.57) |  |
| P-value | 0.029 |  | 0.443 |  |
| **Cerebrovascular disease** |  |  |  |  |
| Population | 215,071 | 386,446 | 36,897 | 65,520 |
| Events, n (%) | 494 (0.22%) | 620 (0.16%) | 124 (0.34%) | 111 (0.17%) |
| Person-years | 1342726 | 2125102 | 248888 | 378288 |
| Crude incident rate/1000 years | 0.37 | 0.29 | 0.50 | 0.29 |
| Age at outcome Median (IQR) | 42.5 (35.8-47.2) | 42 (36.1-47.3) | 43.2 (38.5-48.6) | 42.7 (35.3-47.6) |
| Crude HR (95% CI) | 1.19 (1.06- 1.34) |  | 1.62(1.26-2.00) |  |
| P-value | 0.004 |  | < 0.001 |  |
| Adjusted HR (95% CI) | 1.04 (0.93-1.18) |  | 1.48 (1.14-1.92) |  |
| P-value | 0.480 |  | 0.003 |  |
| **Heart failure** |  |  |  |  |
| Population | 215,332 | 386,749 | 36,935 | 65,579 |
| Events, n (%) | 139 (0.06%) | 138 (0.04%) | 23 (0.06%) | 30 (0.05%) |
| Person-years | 1346027 | 2129232 | 249715 | 379180 |
| Crude incident rate/1000 years | 0.1 | 0.06 | 0.09 | 0.08 |
| Age at outcome Median (IQR) | 44.4 (37.9 - 49) | 41.5 (36.7-46.9) | 45.2 (35.4-48.9) | 39.9 (33.6-45.7) |
| Crude HR (95% CI) | 1.48 (1.17-1.87) |  | 1.10 (0.64-1.90) |  |
| P-value | 0.001 |  | 0.723 |  |
| Adjusted HR (95% CI) | 1.30 (1.03-1.65) |  | 1.03 (0.59-1.79) |  |
| P-value | 0.029 |  | 0.910 |  |
| **Hypertension** |  |  |  |  |
| Population | 212,747 | 383,644 | 36,476 | 65,031 |
| Events, n (%) | 4529 (2.13%) | 5788 (1.51%) | 1060 (2.91%) | 1109 (1.71%) |
| Person-years | 1300272 | 2076051 | 239680 | 369126 |
| Crude incident rate/1000 years | 3.48 | 2.79 | 4.42 | 3.00 |
| Age at outcome Median (IQR) | 41.6 (36.4-46.2) | 41.8 (36.8-46.6) | 42 (37.3 - 47) | 43 (37.7- 47.8) |
| Crude HR (95% CI) | 1.19 (1.14 - 1.24) |  | 1.41 (1.30-1.54) |  |
| P-value | < 0.001 |  | <0.001 |  |
| Adjusted HR (95% CI) | 1.08 (1.04-1.12)* |  | 1.33 (1.22-1.45) * |  |
| P-value | p<0.001 |  | <0.001 |  |
| **Diabetes mellitus** |  |  |  |  |
| Population | 213,480 | 384,466 | 36,613 | 65,201 |
| Events, n (%) | 2412 (1.13%) | 2215 (0.58%) | 582 (1.59%) | 383 (0.59%) |
| Person-years | 1322940 | 2106721 | 244548.3 | 375211.8 |
| Crude incident rate/1000 years | 1.82 | 1.05 | 2.38 | 1.02 |
| Age at outcome Median (IQR) | 40.6 (34.8-45.3) | 41.3 (36.1-46.2) | 40.5 (34.3-46.2) | 42.8 (38.3-47.1) |
| Crude HR (95% CI) | 1.66 (1.56-1.75) |  | 2.25(1.96-2.53) |  |
| P-value | < 0.001 |  | <0.001 |  |
| Adjusted HR (95% CI) | 1.41 (1.33-1.50)# |  | 1.84 (1.61-2.10) # |  |
| P-value | <0.001 |  | <0.001 |  |

Adjusted for age, Townsend quintile of deprivation, body mass index, smoking, lipid medication, Alcohol status, hypertension, diabetes mellitus, current use of combined oral contraceptive, connective tissue disorders, migraine, gestational diabetes mellitus (types 1 and 2), pre-eclampsia, pre-term birth, pelvic inflammatory disease.

*= Adjusted for all above except hypertension

#= Adjusted for all above except diabetes mellitus

**Table S6: Incidence rates and hazard ratios for composite CVD (Sensitivity analyses for menstrual cycle frequency)**

| **Composite CVD** | **Amenorrhoea** | | **Polycystic ovary syndrome** | | **Endometriosis** | |
| --- | --- | --- | --- | --- | --- | --- |
|  | **Exposed** | **Unexposed** | **Exposed** | **Unexposed** | **Exposed** | **Unexposed** |
| Population | 31,096 | 65,484 | 33125 | 64469 | 36457 | 64848 |
| Events, n (%) | 153 (0.49%) | 202 (0.31%) | 182 (0.55%) | 199 (0.31%) | 201 (0.55%) | 199 (0.31%) |
| Person-years | 198450.6 | 377644.7 | 228639.5 | 372663.2 | 245064.4 | 373542.3 |
| Crude incident rate/1000 years | 0.77 | 0.53 | 0.80 | 0.53 | 0.82 | 0.53 |
| Age at outcome Median (IQR) | 43.4 (38.2-49.0) | 43.5 (37.4-48.2) | 43.1 (38.5-48.0) | 43.5 (37.7-48.2) | 43.2 (38.5-48.5) | 43.3 (37.3-48.2) |
| Crude HR (95% CI) | 1.41 (1.14-1.74) |  | 1.40 (1.14-1.72) |  | 1.46 (1.20-1.78) |  |
| P-value | 0.001 |  | 0.001 |  | <0.001 |  |
| Adjusted HR (95% CI) | 1.18 (0.95-1.47) |  | 1.23 (1.01-1.51) |  | 1.24 (1.02-1.52) |  |
| P-value | 0.130 |  | 0.043 |  | 0.035 |  |

Adjusted for age, Townsend quintile of deprivation, body mass index, smoking, lipid medication, Alcohol status, hypertension, diabetes mellitus, current use of combined oral contraceptive, connective tissue disorders, migraine, polycystic ovary syndrome, gestational diabetes mellitus, pre-eclampsia, pre-term birth, pelvic inflammatory disease, endometriosis, fibroids.

| **Composite CVD** | **Current contraceptive use** | | **Fibroids** | |
| --- | --- | --- | --- | --- |
|  | **Exposed** | **Unexposed** | **Exposed** | **Unexposed** |
| Population | 26620 | 48155 | 36729 | 65274 |
| Events, n (%) | 165 (0.62%) | 172 (0.36%) | 204 (0.56%) | 199 (0.30%) |
| Person-years | 177420.8 | 273834.2 | 247286.2 | 376280.8 |
| Crude incident rate/1000 years | 0.93 | 0.63 | 0.83 | 0.53 |
| Age at outcome Median (IQR) | 43.4 (39.3-48.9) | 43.7 (38.3-48.4) | 43.2 (38.5-48.5) | 43.5 (37.3-48.4) |
| Crude HR (95% CI) | 1.40 (1.13-1.74) |  | 1.48(1.21-1.80) |  |
| P-value | 0.002 |  | <0.001 |  |
| Adjusted HR (95% CI) | 1.14 (0.91-1.42) |  | 1.26 (1.03-1.54) |  |
| P-value | 0.259 |  | 0.025 |  |

Adjusted for age, Townsend quintile of deprivation, body mass index, smoking, lipid medication, Alcohol status, hypertension, diabetes mellitus, current use of combined oral contraceptive, connective tissue disorders, migraine, polycystic ovary syndrome, gestational diabetes mellitus, pre-eclampsia, pre-term birth, pelvic inflammatory disease, endometriosis, fibroids.

**Table S7**: **Incidence rates and hazard ratios for cardiometabolic outcomes (Sensitivity analyses for frequent and infrequent cycles separately)**

|  | **Menstrual cycle frequency (short)** | | **Menstrual cycle frequency (Long)** | |
| --- | --- | --- | --- | --- |
|  | **Frequent (exposed)** | **Normal (Unexposed)** | **Infrequent (exposed)** | **Normal (unexposed)** |
| **Composite CVD** |  |  |  |  |
| Population | 16063 | 27976 | 20810 | 37508 |
| Events, n (%) | 125 | 107 | 80 | 95 |
| Person-years | 122864.1 | 183737.4 | 125,437.1. | 193,907.3 |
| Crude incident rate/1000 years | 1.0 | 0.6 | 0.7 | 0.5 |
| Age at outcome Median (IQR) | 44.3 (39.2-49.3) | 44.4 (39.4-49.9) | 41.8 (37.7-46.6) | 42.5 (34.7-46.2) |
| Crude HR (95% CI) | 1.66 (1.28-2.15) |  | 1.24 (0.92-1.67) |  |
| P-value | <0.001 |  | 0.161 |  |
| Adjusted HR (95% CI) | 1.42 (1.09-1.85) |  | 1.06 (0.78-1.45) |  |
| P-value | 0.009 |  | 0.704 |  |
| **IHD** |  |  |  |  |
| Population | 16094 | 28021 | 20839 | 37550 |
| Events, n (%) | 46 | 46 | 31 | 30 |
| Person-years | 123541.7 | 184410.1 | 125, 827.6 | 194497.8 |
| Crude incident rate/1000 years | 0.4 | 0.2 | 0.2 | 0.2 |
| Age at outcome Median (IQR) | 45.9 (41.1-49.8) | 47.8 (42.6-51.4) | 43.0 (39.1-47.8) | 45.0 (39.1-48.1) |
| Crude HR (95% CI) | 1.41 (0.93-2.12) |  | 1.48 (0.89-2.44) |  |
| P-value | 0.102 |  | 0.127 |  |
| Adjusted HR (95% CI) | 1.13 (0.74- 1.72) |  | 1.16 (0.68- 1.97) |  |
| P-value | 0.570 |  | 0.582 |  |
| **Cerebrovascular disease** |  |  |  |  |
| Population | 16079 | 27996 | 20818 | 37524 |
| Events, n (%) | 82 | 55 | 42 | 56 |
| Person-years | 123258.6 | 184112.6 | 125629.7 | 194174.9 |
| Crude incident rate/1000 years | 0.7 | 0.3 | 0.3 | 0.3 |
| Age at outcome Median (IQR) | 43.5 (38.5-49.3) | 43.3 (39.3-49.6) | 41.7 (38.6- 46.2) | 41.6 (33.5-46.2) |
| Crude HR (95% CI) | 2.12 (1.51- 2.99) |  | 1.12 (0.75-1.67) |  |
| P-value | <0.001 |  | 0.583 |  |
| Adjusted HR (95% CI) | 1.88 (1.33-2.67) |  | 1.01 (0.66-1.53) |  |
| P-value | <0.001 |  | 0.980 |  |
| **Heart failure** |  |  |  |  |
| Population | 16094 | 28023 | 20841 | 37556 |
| Events, n (%) | 11 | 16 | 12 | 14 |
| Person-years | 123761.3 | 184559.3 | 125953.5 | 194620.9 |
| Crude incident rate/1000 years | 0.1 | 0.1 | 0.1 | 0.1 |
| Age at outcome Median (IQR) | 47.6 (41.3-49.8) | 38.6 (33.9-48.9) | 39.7 (33.7-46.6) | 40.7 (31.9-45.5) |
| Crude HR (95% CI) | 0.97 (0.45-2.09) |  | 1.27 (0.58-2.74) |  |
| P-value | 0.935 |  | 0.550 |  |
| Adjusted HR (95% CI) | 0.93 (0.42- 2.06) |  | 1.13 (0.50-2.54) |  |
| P-value | 0.858 |  | 0.770 |  |
| **Hypertension** |  |  |  |  |
| Population | 15868 | 27732 | 20608 | 37299 |
| Events, n (%) | 589 | 620 | 471 | 489 |
| Person-years | 118078.5 | 178641 | 121601.2 | 190485.1 |
| Crude incident rate/1000 years | 5.0 | 3.5 | 3.9 | 2.6 |
| Age at outcome Median (IQR) | 43.0 (38.4-47.8) | 43.4 (39.0-48.4) | 40.7 (36.7-45.3) | 42.2 (36.0-46.8) |
| Crude HR (95% CI) | 1.38 (1.23-1.55) |  | 1.44 (1.27-1.64) |  |
| P-value | P<0.001 |  | P<0.001 |  |
| Adjusted HR (95% CI) | 1.37 (1.22-1.54) |  | 1.24 (1.08-1.41) |  |
| P-value | <0.001 |  | 0.002 |  |
| **Diabetes mellitus** |  |  |  |  |
| Population | 15979 | 27860 | 20634 | 37341 |
| Events, n (%) | 239 | 214 | 343 | 169 |
| Person-years | 121590.4 | 182497.1 | 122957.8 | 192714.7 |
| Crude incident rate/1000 years | 2.0 | 1.2 | 2.8 | 0.9 |
| Age at outcome Median (IQR) | 41.4 (34.6-47.8) | 43.2 (39.1-47.2) | 39.8 (34.3-45.1) | 42.0 (37.8-46.6) |
| Crude HR (95% CI) | 1.60 (1.33-1.92) |  | 3.04 (2.53-3.65) |  |
| P-value | <0.001 |  | <0.001 |  |
| Adjusted HR (95% CI) | 1.37 (1.13-1.65) |  | 2.24 (1.85-2.72) |  |
| P-value | <0.001 |  | <0.001 |  |

Adjusted for age, Townsend quintile of deprivation, body mass index, smoking, lipid medication, Alcohol status, hypertension, diabetes mellitus, current use of combined oral contraceptive, connective tissue disorders, migraine, polycystic ovary syndrome, gestational diabetes mellitus (types 1 and 2), pre-eclampsia, pre-term birth, pelvic inflammatory disease, endometriosis, fibroids.

*= Adjusted for all above except hypertension

#= Adjusted for all above except diabetes mellitus

| A  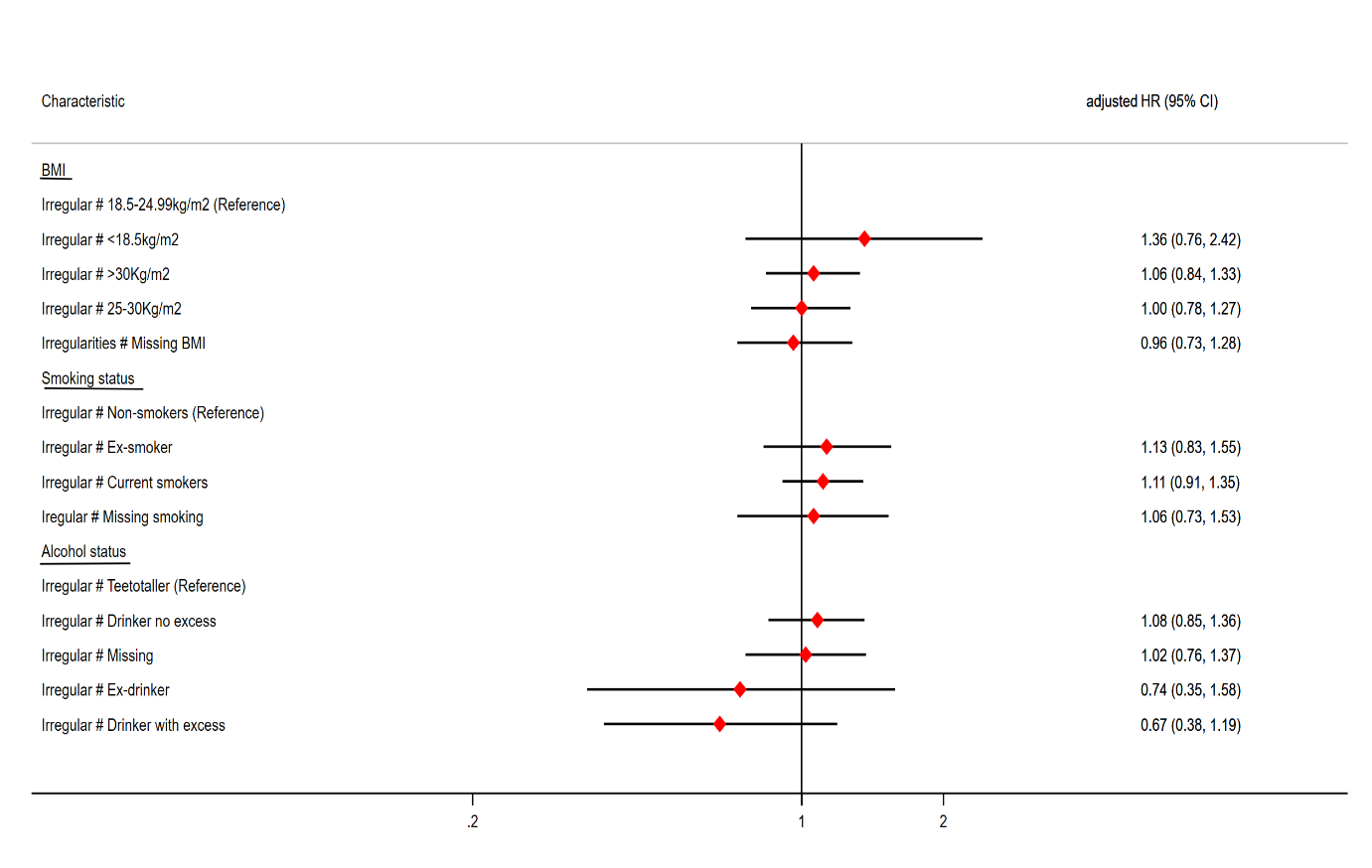 |
| --- |
| B  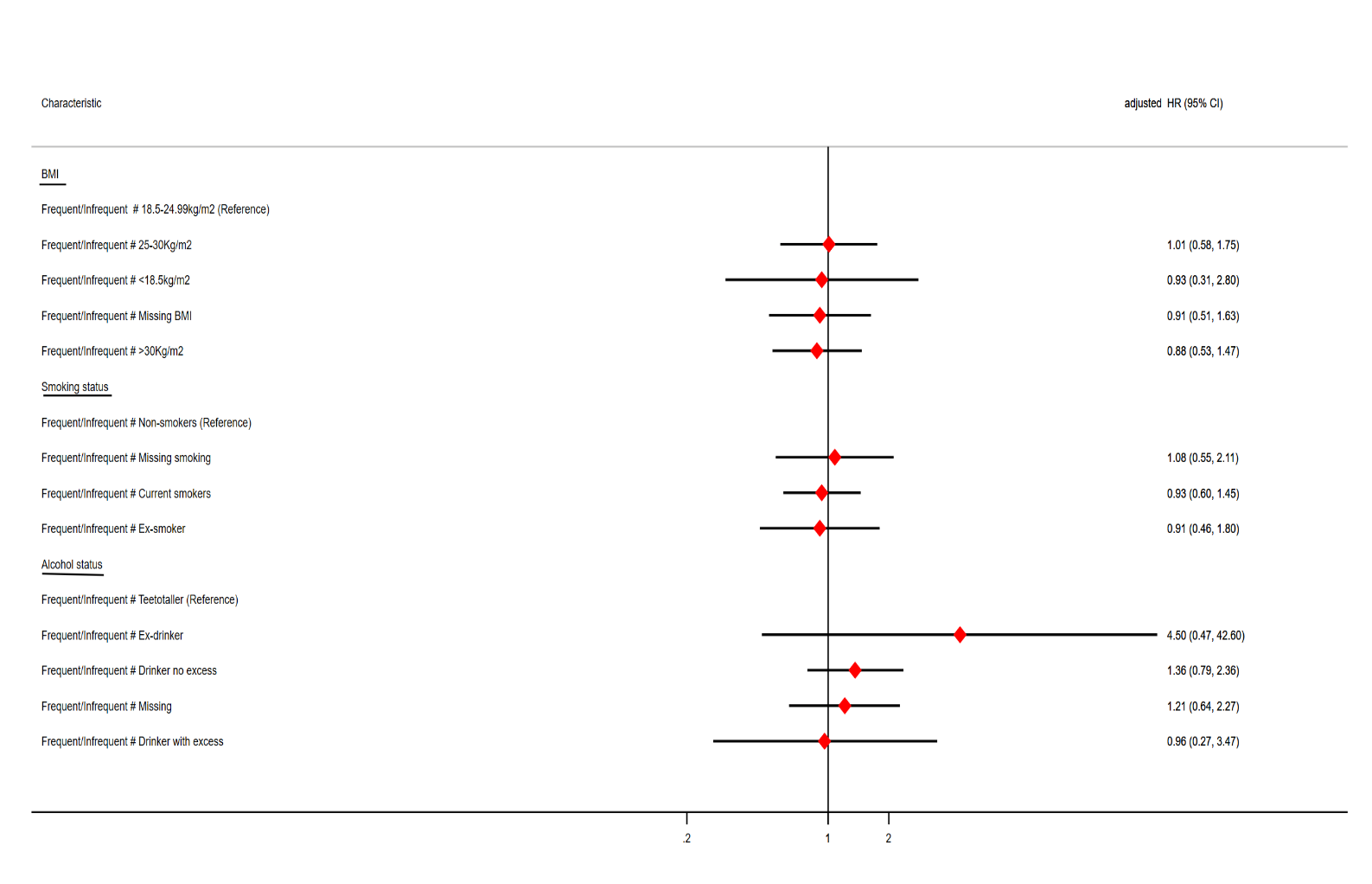 |

Figure S2: Interaction between (A) irregular menstrual cycles and (B) frequent or infrequent menstrual cycles and lifestyle factors (Body mass index, smoking, and alcohol use)

**Table S8: Summary of selected existing literature examining the association between menstrual characteristics and cardiometabolic outcomes.**

| **Author year** | **Objective** | **Study design & population** | **Exposure** | **Confounder adjustment** | **Outcome & results (Multivariable analyses)** |
| --- | --- | --- | --- | --- | --- |
| Iliodromiti & Nelson (34)  2017  UK | To assess whether irregular menstrual cycles are associated with CVD events (morbidity and mortality) | Prospective cohort UK biobank  40,896 Non menopausal women aged 50 years and below. | Menstrual cycle regularity reported as irregular cycles or cycles longer than 34 days (N= 8835) versus regular cycles (N=32061) | Adjusted for multiple confounders (not provided) | ***Irregular menstrual cycles***  Fatal and non-fatal CVD events HR 1.05 (95% CI, 0.91-1.20)  Fatal and non-fatal IHD events HR 0.88(95% CI, 0.58-1.34)  Fatal and non-fatal cerebrovascular events HR 0.85 (95% CI, 0.47 to 1.51) |
| Wang et al. (9)  **2022**  USA | To investigate whether menstrual cycle characteristics across the reproductive lifespan are associated with CVD. How strongly do hypercholesterolemia, chronic hypertension mediate the association | Prospective cohort study. (Nurses’ Health Study II)  80 630 female Nurses from the US  Follow-up 1993-2017  Menstrual cycle characteristics (regularity and length reported for ages 14-17 years and 18-22 years at enrolment 1989 and in 1993 at ages 29-46 years | Menstrual cycle regularity  Very regular (3-4 days before or after expected)  Regular (within 5-7 days)  Usually, irregular  Always irregular  No periods  Menstrual cycle length  < 12 days  21-25 days  26-31 days  32-39 days  40-50 days  >50 days or too irregular to estimate | Age, race & ethnicity, age at menarche,  parental history of CVD BMI, time-varying menopausal status and hormone usage, hormone therapy use, parity, regular aspirin use, physical activity, smoking status, Alternative Healthy Eating Index diet | Fatal and Non-fatal composite CVD (MI, revascularization, CABG, PCI, and stroke)  **Cycle regularity (Fully adjusted model 2)**  *14-17 years:*  Always irregular or no period (HR 1.15; 95% CI, 0.99-1.34)  Usually irregular (HR 1.05; 0.99-1.34)  *18-22 years:*  Always irregular or no period (HR 1.36; 95% CI, 1.06-1.75)  Usually irregular: (HR 1.08; 95% CI 0.85-1.37)  *29-46 years:*  Always irregular or no period (HR 1.40; 95% CI, 1.14-1.71)  Usually irregular: (HR 1.26; 95% CI ,1.06-1.49)  **Cycle length (Fully adjusted model 2)**  18-22 years*:*  > 40 days or too irregular (HR 1.44; 95% CI, 1.13-1.34)  32-39 days: (HR 1.06; 95 CI, 0.86-1.31)  29-46 years:  *>* 40 days or too irregular (HR 1.30; 95% CI, 1.09-1.57)  32-39 days: (HR 1.05; 95% CI 0.89-1.24) |
| Wang et al. (8)  **2020**  USA | To determine whether irregular or prolonged menstrual cycles are associated with all-cause and cause-specific premature mortality (70 years of age). | Prospective cohort study. (Nurses’ Health Study II)  79505 female Nurses from the US  Follow-up 1993-2017  Menstrual cycle characteristics (regularity and length reported for ages 14-17 years and 18-22 years at enrolment 1989 and in 1993 at ages 29-46 years | Menstrual cycle regularity  Very regular (3-4 days before or after expected)  Regular (within 5-7 days)  Usually, irregular  Always irregular  No periods  Menstrual cycle length  < 12 days  21-25 days  26-31 days  32-39 days  40-50 days  >50 days or too irregular to estimate | Age, menopausal status, age at menarche, race, family history of myocardial infarction, stroke, diabetes, baseline hypertension, cholesterol levels, parity, alcohol consumption, BMI, physical activity, smoking status, alternative health eating index. | Premature (< 70 years) CVD  **Cycle regularity (Fully adjusted model 2)**  *14-17 years:*  Irregular or no period (HR 1.11; 95% CI, 0.76-1.61)  *18-22 years:*  Irregular or no period (HR 1.24; 95% CI, 0.70-2.18)  *29-46 years:*  Irregular or no period (HR 1.59; 95% CI, 1.04-2.45)  **Cycle length (Fully adjusted model 2)**  18-22 years*:*  > 32 days or too irregular (HR 1.16; 95% CI, 0.67-2.04)  29-46 years:  > 32 days or too irregular (HR 1.46; 95% CI, 0.99-2.16) |
| Wang et al. (35)  2020  USA | To examine the association between menstrual cycle characteristics and risk of type 2 diabetes mellitus | Prospective cohort (Nurses Health II) study  75,546 premenopausal US female nurses aged 29-46 years at baseline.  Menstrual cycle characteristics (regularity and length reported for ages 14-17 years and 18-22 years at enrolment 1989 and in 1993 at ages 29-46 years | Menstrual cycle irregularity reported as regular (within 5-7 days of expected period), usually irregular, always irregular, no period.  Menstrual cycle length defined as usual (< 21 days), 21-25 days, 26-31 days, 32-39 days, 40-50 days, > 50 days or too irregular to estimate | Age, age at menarche, ethnicity, family history of diabetes, menopausal status, menopausal hormone use, parity, household income, oral contraceptive use, alcohol consumption, BMI, physical activity, smoking status, and Alternative Healthy Eating Index diet quality score (quintiles) | Outcome Type 2 diabetes mellitus  **Irregular menstrual cycles (Fully adjusted model 2)**  14-17 years  Always irregular or no period: (HR 1.32; 95%CI, 1.22-1.44)  Usually irregular: (HR 1.15; 95% CI 1.05-1.25)  Ages18-22 years  Always irregular or no period (HR 1.41 95%CI, 1.23-1.62)  Usually irregular (HR 1.22; 95% CI, 1.07-1.39)  Ages 29-46 years  Always irregular or no period (HR1.66 95%CI, 1.49-1. 84)  Usually irregular (HR 1.35; 95% CI 1.23-1.48)  **Cycle length (Fully adjusted model 2)**  Ages 18-22 years  >40 days: (HR 1.37; 95% CI 1.19-1.57)  32-39 days: (HR 1.18; 95% CI 1.06-1.33)  Ages 29-46  >40 days: (HR 1.50; 95% CI 1.36-1.65)  32-39 days: (HR 1.37; 95% CI 1.36-1.65) |
| Kiconco et al. (36)  2021 Australia | To evaluate risk of heart disease and diabetes mellitus in women with irregular menstrual cycles compared to those with regular menstrual cycles. | Longitudinal cohort study of 13714 women aged 45-50 years at baseline | Menstrual cycle irregularity (N=1048) and regular menstrual cycle (N= 12135) | age, country of birth, education level, occupation, marital status, alcohol consumption, physical activity, oral contraceptive pill use  use, hormonal replace therapy use, and body mass index | ***Irregular menstrual cycles***  Heart disease (myocardial infarction and angina) HR 1.20 (95% CI, 1.01-1.43)  Diabetes mellitus HR 1.17 (95% CI, 1.00-1.38) |
| Dovom et al. (37)  2016  Iran | To evaluate the association between history of irregular menstrual cycles and metabolic disorders | Prospective cohort (Tehran lipid and glucose study) of 2128 Iranian women aged 18-49 years at baseline.  Duration of follow-up: 15 years | Menstrual cycle regularity reported as regular as regular versus irregular | age, BMI, parity, FBS, BS-2hr, family history of diabetes mellitus, menstrual status for Diabetes mellitus | ***Irregular menstrual cycles***  Diabetes mellitus: HR 1.73 (95% CI, 1.14-2.64)  Hypertension: HR 1.26(95% CI, 0.89-1.80) |
| Solomon et al. (38)  2002  USA | To evaluate the risk of CHD and stroke associated with history of irregular menstrual cycles | Prospective cohort study (Nurses Health II) study.  82,349 female nurses from the USA aged 25-35 years at baseline.  Follow-up from 1982-1996 | Menstrual cycle regularity reported via questionnaire as very regular, usually regular, very irregular | age, BMI, cigarette smoking, menopausal status, HRT use, parental history of MI, parity alcohol use, aspirin use, multivitamin, use of vitamin E supplements, physical activity level, history of oral contraceptive use. | ***Very irregular menstrual cycle***  Composite CVD fatal and non-fatal  aRR 1.46 (95% CI, 1.23–1.74)  Fatal and non-fatal CHD aRR 1.53 (95% CI, 1.24–1.90)  Non-fatal CHD aRR 1.38 (95% CI, 1.06-1.80)  Fatal CHD aRR 1.88 (95% CI, 1.32-2.67) Overall (fatal and non-fatal) stroke aRR, 1.30; 95% CI (0.97–1.74)  *Usually, irregular*  Composite CVD aRR 1.17(95% CI, 1.03-1.33)  Total CHD aRR 1.22 (95% CI, 1.04–1.44)  Non-fatal aRR 1.27 (95% CI, 1.05–1.54)  Fatal CHD aRR 1.11 (95% CI, 0.82–1.50)  *Usually, regular*  Total CHD aRR 1.11 (95% CI,0.82–1.50)  Non-fatal CHD aRR 0.96 (95% CI, 0.82–1.12)  Fatal CHD aRR 1.12 (95% CI, 0.90–1.40) |
| Solomon et al. (39)  2001  USA | To evaluate the risk of type 2 diabetes mellitus in women with long or irregular menstrual cycles | Prospective cohort study. 101, 073 women aged 18-22 years at baseline  Duration of follow-up: 8 years | Menstrual cycle characteristics regularity reported as very regular (within 3 days), regular, usually irregular, always irregular, no period.  Menstrual cycle length reported as <21 days, 21-25 days, 26-31 days, 32-39 days, 40-50 days, >50 days, or too irregular to estimate) | Age, time period, BMI, smoking, family history of diabetes mellitus, physical activity level, and duration of oral contraceptive use. | ***Irregular menstrual cycles or no period***  Usual cycle length in days.  26-31 days: RR 1.0 (Reference)  < 21 days: RR 1.50 (0.70-3.19)  21-25 days: RR 1.18 (0.87-1.58)  32-39 days: RR 1.03 (0.79-1.33)  >= 40 days or highly irregular: RR 2.08 (1.61-2.66) |
| Gast et al. (40)  2010  Netherlands | To examine whether long and irregular cycles are associated with future risk of type 2 DM and CHD | Prospective cohort study (The Netherlands Prospect and MORGEN cohort)  23,571 women aged 30-40 years at baseline. | Menstrual cycle characteristics defined as irregular, regularly short (<26 days), regularly normal (27 -29 days),  regularly long (30  days)  Cycle length was defined as long (≥ 30 days) or short (≤ 26 days) | Age, BMI waist–hip ratio, physical activity, smoking,  education level, ever use of OCs, HT, oophorectomy, and  menopausal status, | ***Irregular menstrual cycles***  Coronary Heart disease HR 1.28 (95% CI, 1.05-1.56)  Type 2 diabetes mellitus HR 1.21 (95% CI 0.96- 1.54) |
